# From Powerhouse to Perpetrator—Mitochondria in Health and Disease

**DOI:** 10.3390/biology8020035

**Published:** 2019-05-11

**Authors:** Nima B. Fakouri, Thomas Lau Hansen, Claus Desler, Sharath Anugula, Lene Juel Rasmussen

**Affiliations:** 1 Laboratory of Molecular Gerontology, National Institute on Aging, National Institutes of Health, Baltimore, MD 21224, USA; nima.borhanfakouri@nih.gov; 2 Center for Healthy Aging, Department of Cellular and Molecular Medicine, University of Copenhagen, 2200 Copenhagen, Denmark; tlhansen@sund.ku.dk (T.L.H.); cdesler@sund.ku.dk (C.D.); sharath@sund.ku.dk (S.A.)

**Keywords:** mitochondria, cancer, nucleotide metabolism, DNA damage, NAD^+^

## Abstract

In this review we discuss the interaction between metabolic stress, mitochondrial dysfunction, and genomic instability. Unrepaired DNA damage in the nucleus resulting from excess accumulation of DNA damages and stalled replication can initiate cellular signaling responses that negatively affect metabolism and mitochondrial function. On the other hand, mitochondrial pathologies can also lead to stress in the nucleus, and cause sensitivity to DNA-damaging agents. These are examples of how hallmarks of cancer and aging are connected and influenced by each other to protect humans from disease.

## 1. Introduction

It has been almost two decades since Hanahan and Weinberg for the first time classified the hallmarks of cancer [1]. Ten years later, they updated that list and introduced genomic instability and dysregulation of cellular energetics and mitochondrial function as emerging hallmarks [2]. Recently, both genomic instability and mitochondrial dysfunction are considered as two of the key hallmarks of aging [3]. Both of these have been implicated in several pathologies, as reviewed in References [4,5,6]. There are other hallmarks that are common between cancer and aging, such as epigenetic changes and altered cellular communication. Moreover, other hallmarks are in opposition to each other in cancer and aging. These include the dysregulation of apoptosis and senescence, which are stimulated in aging cells and suppressed in cancer cells [2,3]. Whether we can consider cancer as the disease of aging is a topic that is beyond the scope of this review, but age is the largest risk factor in the development of cancer [7].

One of the key questions that remain to be answered is, how are these hallmarks are connected and influenced by each other [3]? As these pathologic cellular changes occur gradually, understanding the connection between them would help to develop more effective therapeutic strategies to treat cancer or rather prevent it.

**Figure 1:**
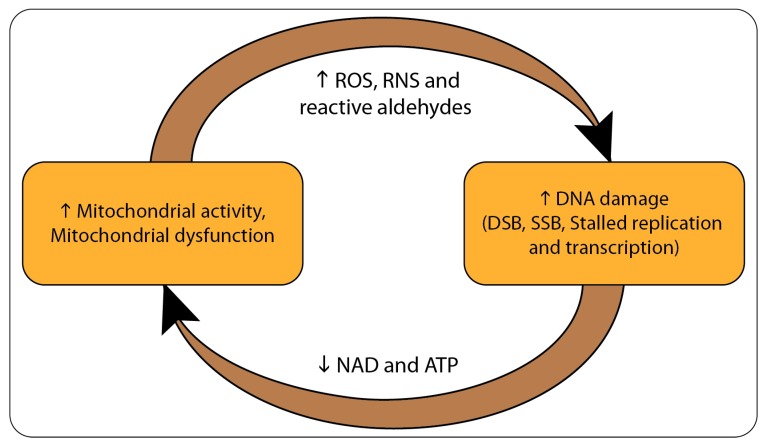
Illustration of the mitochondrial-nuclear interactions in aging or cancer. Abnormal metabolism and/or metabolic defects lead to metabolic stress and mitochondrial dysfunction. This is followed by the increased generation of reactive oxygen and nitrogen species (ROS and RNS) as well as reactive aldehydes. These reactive species can react and damage macromolecules such as proteins and DNA. Damage to DNA causes genomic instability via stalled replication and transcription, and the generation of double- and single-strand breaks (DSBs and SSBs, respectively) within the genome. Increased activities of the DNA damage response (DDR) deplete cells of key cellular substrates and cofactors, mainly ATP and NAD^+^. This generates a positive feedback that enhances metabolic stress and mitochondrial dysfunction.

It has long been known that byproducts of cellular metabolism such as reactive oxygen and nitrogen species (ROS and RNS) can damage cellular components and macromolecules including DNA [8,9]. Damage to DNA can have severe effects on cells by blocking replication, transcription, generation of DNA double and single strand breaks, as well as chromosome rearrangements [10,11]. Activation of the DNA damage response (DDR) following DNA damage is an energy-demanding process [12], and can deplete cells of substrates such as NAD^+^ and ATP, which can in turn lead to additional metabolic stress and mitochondrial dysfunction (Figure 1) [13,14,15].

In this review, we aim to discuss the interaction between metabolic stress and mitochondrial dysfunction with genomic instability. Stress in the nucleus, such as the accumulation of DNA damage and stalled replication, negatively affects metabolism and mitochondrial function [13,14,15,16,17], while mitochondrial pathologies lead to stress in the nucleus [18] and cause sensitivity to DNA-damaging agents [19], as reviewed by Desler et al. in 2012 [20].

First, we explain how the accumulation of DNA damages and the activation of the DDR leads to mitochondrial dysfunction. Next, we explain how the dysregulation of mitochondrial function and metabolism contributes to the epigenetic changes, imbalanced dNTP pools, and genomic instability.

## 2. From DNA Damage to Mitochondrial Dysfunction

**Figure 2:**
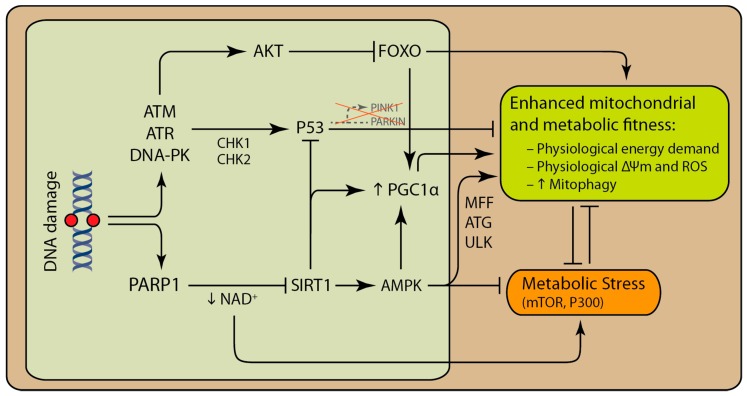
Core DNA damage response proteins are able to influence mitochondrial activity and quality control via multiple pathways. Abnormal DNA structure and certain genomic lesions, such as strand breaks, activate poly (ADP-ribose) polymerase (PARP) enzymes, mainly PARP1. Chronic activation of PARP1 can deplete the cell of NAD^+^, which is a rate-limiting substrate for SIRT1. SIRT1 together with AMP-activated kinase (AMPK) can enhance metabolism and mitochondrial function by enhancing mitochondrial biogenesis and mitophagy. Activation of ataxia telangiectasia mutated (ATM), Rad3-related (ATR) and DNA-dependent protein kinase (DNA-PK) following DNA damage can promote the activation AKT and P53. Activation of AKT rewires cellular metabolism by inhibiting Forkhead box (FOXO) enzymes. Deacetylation of P53 by SIRT1 targets P53 for degradation. Decrease in SIRT1 activity stabilizes P53. P53 decreases mitophagy via inhibition of PTEN-induced kinase 1 (PINK1) and PARKIN transcription. SIRT1 promotes AMPK activity indirectly. Decrease in SIRT1 activity is followed by the decrease in activated AMPK.

Activation of the main components of the DDR, including poly (ADP-ribose) polymerase (PARP) enzymes (mainly PARP1 and PARP2) as well as ataxia telangiectasia mutated (ATM) [21], DNA-dependent protein kinase (DNA-PK) [22,23] and P53 [24,25,26,27], is able to influence mitochondrial function and cellular metabolism. Chronic activation of PARP1 negatively affects cellular physiology and mitochondrial function [28]. Activation of ATM, DNA-PK, and P53 can influence mitochondrial and cellular metabolism to promote either cell survival or death (Figure 2). Here, we briefly describe how each of these enzymes are able to influence mitochondrial function.

## 3. PARP Modulates Mitochondrial Function and Cellular Metabolism

PARPs are a group of enzymes (16 in mice and 17 in humans) that are the main constituent of the cellular stress response [29]. PARPs cleave NAD^+^ to nicotinamide (NAM) and ADP-ribose (ADPR), and the ADPR is subsequently transferred to certain amino acids within the target protein. The attachment of poly ADP-ribose (PAR) to the target proteins is referred to as PARylation, and it can affect protein–protein and protein–DNA interaction as well as protein localization [30]. PAR has a short half-life and is degraded almost directly after its formation by the activity of the PAR-degrading enzyme, poly(ADP-ribose) glycohydrolase (PARG) [31]. PARylation modulates several key cellular processes such as chromatin structure, transcription, translation, cell cycle, DNA repair, mitochondrial homeostasis, apoptosis, and metabolism [29,32]. PARP1 is activated by several mechanisms, including mono(ADP-ribosyl)ation, phosphorylation, and acetylation [29]. PARP1 possesses a DNA-binding domain that recognizes abnormal DNA structures such as gapped DNA, single- and double strand breaks, cruciform structures, and nucleosome linker DNA [33,34]. In the initial steps of the repair, PARP1 PARylates histones and facilitates the chromatin relaxation that provides more space for the recruitment of DNA repair proteins. Subsequent PAR generation recruits the DNA repair proteins via their PAR-binding domains [35,36]. Under mild genotoxic stress, PARP activation results in repair and survival. However, in response to DNA damage, PARP hyper-activation results in decrease in NAD^+^ and ATP levels, mitochondrial dysfunction, and eventually cell death [32,35,37].

## 4. DNA Damage can Activate Both Pro-Survival and Pro-Death Pathways That Involve the Mitochondria

DNA damage and DDR can activate pathways that promote cell survival or death depending on the extent and type of DNA damages [38]. In addition to PARP1, other immediate sensors of DDR are enzymes belonging to the superfamily of phosphatidylinositol 3-kinase-related kinases (PIKKs), including ATM, ataxia telangiectasia and Rad3-related (ATR), and DNA-PK. Activated ATR and ATM, in turn, activate P53 through CHK1 and CHK2, respectively. Apart from preventing cell cycle progression and the recruitment of DNA repair proteins, these enzymes can modulate mitochondrial function and survival [23,39]. ATM, ATR, and DNA-PK are able to promote survival via the direct phosphorylation of AKT (also known as protein kinase B, PKB) independently of growth factor signaling [22,40,41,42,43]. However, the mechanism of this interaction is not fully understood [44]. This is particularly interesting, as in many cancer cells, the AKT is activated independently of growth factors [45]. Activated AKT stimulates glucose uptake and ATP production through glycolysis, one of the main hallmarks of cancer, also known as the Warburg effect [46]. In addition, activated AKT inhibits Forkhead box (FOXO) transcription factors [47,48]. FOXO proteins regulate the expression of key genes that are involved in mitochondrial biogenesis and homeostasis, such as peroxisome proliferator-activated receptor gamma co-activator 1alpha (PGC1a) and PTEN-induced kinase 1 (PINK1). Decrease in FOXO activity results in the decrease of mitochondrial biogenesis, mitophagy, autophagy, and lipolysis [49].

## 5. Mito-Nuclear Signaling in Aging and Cancer

**Figure 3:**
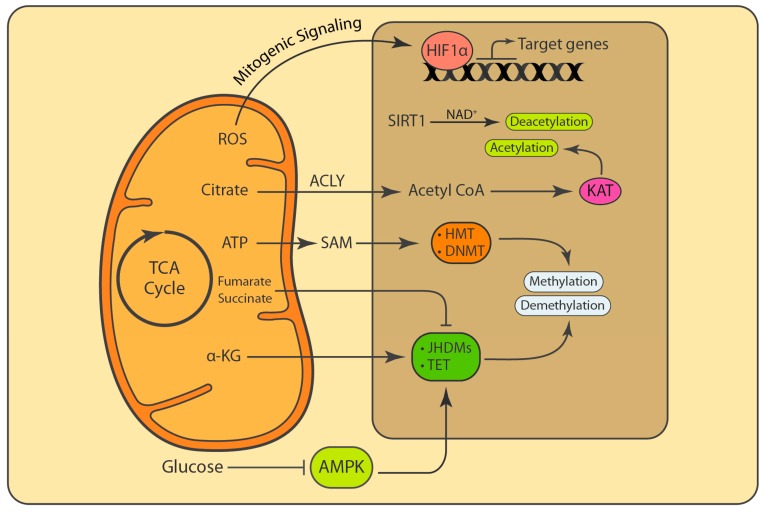
Mitochondrial function and metabolites influence nuclear processes. Mitochondrial ROS and secondary metabolites act as signaling molecules and cofactors that regulate fundamental nuclear processes. Mitochondrial ROS that are released into the cytosol stabilize hypoxia-inducible factor 1- α (HIF1α) and activate the transcription of genes that are involved in proliferation. Citrate generated through the TCA cycle is released into the cytosol and in the nucleus. It is further converted into acetyl-coenzyme A (acetyl CoA) that is used for the acetylation of target proteins. Through a reverse reaction, SIRT1 uses NAD^+^ to deacetylate the target proteins. S-adenosylmethionine (SAM) is a methyl donor that is generated from methionine and ATP in the cytosol. Demethylases such as Jumonji C (JMJC) family members and the ten-eleven translocation (TET) methylcytosine hydroxylases use α-ketoglutarate (α-KG) as cofactor to remove methyl groups from proteins and DNA. AMPK stimulates the activity of TET enzymes, and thus the inhibition of AMPK by glucose impairs the function TET enzymes. The accumulation of fumarate and succinate due to impaired fumarate hydratase (FH) and succinate dehydrogenase (SDH) can inhibit α-KG-dependent demethylases and even cause defects in homologous recombination (HR) DNA repair.

For a long time, it was believed that mitochondria are regulated from the nucleus by the nuclear genome, and changes in mitochondria follow changes in the nucleus [50,51]. However, during recent years, accumulative evidence suggest that mitochondria and mitochondrial metabolites can influence nuclear processes and gene expression in response to various stimuli and environmental cues [52,53,54]. Mitochondrially generated ROS and intermediate metabolites are essential for several processes, including proliferation, epigenetic modifications, and post-translational modifications [54,55]. In addition, mitochondria contribute to genomic stability by replenishing dNTP pools for replication and repair of the genome (Figure 3) [56,57]. In this section we will discuss nuclear processes that are dependent on mitochondrial function and intermediate metabolites.

## 6. Mitochondrial ROS Are Involved in Signaling and Determine Cell Fate

The mitochondrial electron transport chain (ETC) generates reactive oxygen species (ROS) as the byproduct of oxidative phosphorylation (OXPHOS) from different complexes, though mainly complexes I, II, and III. While complexes I and II exclusively create O_2_∙ in the mitochondrial matrix, complex III produces O_2_∙ in both the matrix and intermembrane space [58]. However, the ROS that are released outside of the matrix are converted into H_2_O_2_ by cytosolic superoxide dismutase 1 (SOD1) and participate in mitochondrial signaling through reversible cysteine oxidation [59]. Mitochondrially produced ROS can serve as second-messenger molecules. Mitochondrial ROS (mtROS) are required for the stabilization of HIFα (hypoxia-inducible factor 1-α) and the activation of downstream pathways that promote proliferation [60]. 

It is possible that the type of cell and energy demand determine the effect of ROS and mtDNA mutation over the cell. Cells that mostly rely on glycolysis will probably not be affected by mutation in mtDNA under physiological conditions. During the exposure to stress and stimuli, however, these cells might not be able to trigger an adaptive response to an increase in demand for ATP and NAD^+^ [61]. During stress and increased energy demand, cells respond by boosting cellular respiration and mitochondrial activity to provide the cells with ATP and NAD^+^ [12,62]. This is accompanied by increased mitochondrial membrane potential and ROS above the physiological level [61,63]. Increased ROS cause damage to macromolecules such as DNA, proteins, and lipids, which can cause cell death or malignancy, as reviewed by Sies et al. in 2017 [64]. The inability to enhance ATP production in response to stress and increased energy demand probably contributes to cellular deterioration during aging [65]. 

In contrast to increased ROS generation, a decrease in ROS in metabolically active or proliferative tissues interferes with cellular metabolism or proliferation that can promote senescence, as reviewed by Diebold and Chandel in 2016 [66].

Hematopoietic stem cells (HSCs) represent an excellent example for both situations [67]. HSCs are mainly quiescent, and they rely on glycolysis for ATP production [68]. While low levels of ROS prevent the proliferation of HSCs and maintain their quiescent state, stress and increased energy demand promote a shift toward ATP production by mitochondria and OXPHOS. This is accompanied by increased ROS and proliferation [67]. Chronic stress followed by enhanced mitochondrial activity and ROS generation leads to the depletion of HSCs, which is one of the hallmarks of aging [69,70,71].

## 7. Mitochondria Influence Post-Translational Modifications (PTMs) and Epigenetic Marks

Reversible acetylation and methylation are two frequently employed post-translational modifications that regulate a variety of protein functions, protein stability, gene expression [72], as well as DNA repair [73]. Epigenetic changes are also one of the main hallmarks of both aging and cancer [2,3]. The modifications include alterations in DNA methylation, modifications of histones, and chromatin remodeling. While DNA methylations show similar patterns in aging and cancer, the histone modifications show distinct patterns, as reviewed by Zane et al. in 2014 [74].

The multiple enzymatic systems assuring the generation and maintenance of epigenetic patterns include DNA methyltransferases, histone acetylases, deacetylases, methylases, and demethylases, as well as protein complexes implicated in chromatin remodeling. Most PTMs, such as phosphorylation, acetylation, methylation, and O-linked N-acetylglucosamine modification (O-GlcNAcylation), require metabolites as substrates [75]. If not all, the majority of substrates required for PTMs are intermediate metabolites generated by mitochondria [54]. Chromatin modifiers use metabolic intermediates as cofactors or substrates, but are also regulated by their availability. These metabolites include NAD^+^ for deacetylation, acetyl-CoA for histone acetylation, S-adenosylmethionine (SAM) for histone as well as DNA methylation, and α-ketoglutarate (α-KG) for demethylation [54].

Lysine acetyltransferases (KATs) add acetyl groups to proteins while lysine deacetylases (KDACs) remove acetyl groups from proteins [76,77]. KATs such as GCN5, CBP/p300, and MYST use acetyl-coenzyme A (acetyl CoA) as an acetyl group donor for protein acetylation [78]. KDACs are classified into two groups with different catalytic mechanisms: Zn^2+^-dependent histone deacetylases (HDAC1-11) and NAD^+^-dependent deacetylases (SIRT1-7) [76,77]. Acetyl CoA is generated in mitochondria and converted to citrate through the TCA cycle. Citrate can then be exported from mitochondria. In the cytoplasm and nucleus, citrate is converted back into acetyl CoA via the function of ATP-citrate lyase (ACLY). Citrate is the major source of acetyl CoA in the cytoplasm and the nucleus. Depletion of mtDNA and the subsequent decrease in NAD negatively affect the TCA cycle and lead to a decrease in histone acetylation. This can be rescued by the restoration of electron flow and TCA cycle [60,79]. The degree of acetylation directly correlates with the availability of cofactors such as acetyl CoA and NAD^+^. While increased nuclear acetyl CoA promotes increased acetylation and the formation of euchromatin, an increase in NAD^+^ promotes deacetylation and the formation of heterochromatin, resulting in a decrease of gene expression [79]. MYC and AKT stimulate nutrient uptake and promote acetyl CoA production via ACLY. AKT can directly phosphorylate and activate ACLY to maintain the acetyl CoA levels, regardless of glucose concentrations [80].

Another key chromatin modification that is strongly interconnected with metabolism is methylation [54,81]. Methylation is regulated by S-adenosylmethionine (SAM) abundance, whereby SAM serves as a universal methyl donor, synthesized from methionine and ATP by methionine adenosyltransferases (MATs) [54,81]. Histone and DNA methylation is removed by demethylases such as Jumonji C (JMJC) family members and the ten-eleven translocation (TET) methylcytosine hydroxylases, which use a dioxygenation reaction that requires Fe^2+^, O_2_, and α-ketoglutarate (α-KG) as cofactors [82]. α-KG is generated in the TCA cycle via the catabolism of glucose and glutamine. The function of lysine-specific histone demethylase 1A (LSD1; KDM1A) and LSD2 (KDM1B), which catalyze an amine oxidation reaction, is dependent on flavin adenine dinucleotide (FAD) [83,84]. 

The dysregulation of the TCA cycle and cellular metabolism affect PTMs, epigenetic changes, and choice of DNA repair pathway. Accumulation of succinate or fumarate, which occurs respectively in tumors deficient for succinate dehydrogenase (SDH) or fumarate hydratase (FH), similarly inhibits α-KG-dependent enzymes, leading to defects in homologous recombination (HR) DNA repair [53]. Changes in nutrient availability can directly affect chromatin modifications. The tumor suppressor ten-eleven translocation (TET) protein family of dioxygenases (TET1, TET2, and TET3) converts a 5mC DNA methylation to a hydroxymethylation, 5hmC [85]. AMP-activated kinase (AMPK) phosphorylates TET2 and stabilizes this tumor suppressor protein. However, increase in blood glucose levels impairs AMPK activity, which leads to destabilization of TET2 and the subsequent dysregulation of 5mCs and 5hmCs [86]. 

## 8. Regulation of dNTP Pools

In humans, nucleotide levels are maintained by the nucleotide salvage and/or de novo synthesis of ribo- and deoxyribonucleotide triphosphates (rNTPs and dNTPs).

It is generally accepted that the levels and especially the relative balance of the cytosolic dNTP pools have great influence on the replication, repair, and stability of the nuclear genome [87,88,89,90,91,92]. The efficiency and fidelity of most, if not all, polymerases and many repair enzymes are affected by the level of substrate nucleotides [87]. Too low a concentration of a nucleotide results in poor incorporation frequency, and a level which is too high risks the misincorporation of the high-concentration nucleotide [93,94,95]. Therefore, it is of no surprise that the process of synthesizing dNTPs in the right concentrations is governed by a generous amount of regulation, feedback loops, and redundancy [96,97].

Mitochondrial dysfunction, due to mutations in the mitochondrial genome or a decrease in the mitochondrial DNA (mtDNA) copy number, is associated with a poor prognosis of many types of cancer [98,99,100,101,102], also reviewed by Chatterjee et al. in 2006 [103]. The accumulation of mutations in mtDNA or impeded ETC have even been associated with tumor aggressiveness [99,101,104,105,106]. We have previously shown a relationship between mitochondrial respiration and the regulation of cytosolic dNTP pools, and demonstrated a co-occurring decrease of chromosomal stability [51]. 

The de novo synthesis of nucleotides is split up into purine and pyrimidine synthesis, which go through two distinct pathways.

Despite having separate synthesis pathways, both purine and pyrimidine-based ribonucleotides occupy a central role in cellular metabolism. In addition to being basal components of DNA and RNA, they function as phosphate donors in the transport of cellular energy and participate in enzymatic reactions as well as intracellular and extracellular signaling [107].

Both the salvage and the de novo synthesis pathways utilize an activated sugar intermediate: 5-phosphoribosyl-1-pyrophosphate (PRPP). PRPP is generated by the action of PRPP synthetase and is utilized in both purine and pyrimidine synthesis [108]. The purine nucleotides are synthesized from PRPP, through inosine 5-monophosphate (IMP), and further into AMP and GMP, through two separate but allosterically regulated pathways. Pyrimidines, on the other hand, originate from the precursor pyrimidine nucleotide, UMP, which is used to synthesize all the cellular ribosyl and deoxyribosyl pyrimidines including UTP, CTP, dUMP, and dTTP [108]. A key catalytic enzyme in this process is the dihydroorotate dehydrogenase (DHODH) [109], located in the inner mitochondrial membrane and functionally codependent with the OXPHOS [110]. The oxidation of dihydroorotate by DHODH, to form orotate, is a bottleneck reaction of the de novo synthesis of pyrimidines and is electrochemically coupled with the reduction of ubiquinone to ubiquinol [56,111,112,113]. Mitochondrial respiration can modulate the nucleotide synthesis at two separate steps. A decrease of the mitochondrial respiration is therefore linked to an inhibition of the DHODH enzyme, which in turn modulates the synthesis of pyrimidines [51,114]. Furthermore, the activity of the RNR complex is regulated by the binding of ATP to its active site and inhibited by dATP. By affecting the levels of cytosolic ATP, the mitochondria can influence the activity of RNR, and hence the levels of dNTPs. Furthermore, RNR is allosterically regulated by the relative levels of individual NTPs and dNTPs, and so the de novo synthesis of dNTPs and their relative balance is highly dependent on the mitochondrial supply of pyrimidines and ATP [114,115,116].

The cytosolic pool of dNTPs, which supplies the replication of the nuclear genome, is cell cycle-regulated. Synthesis is initiated at the beginning of the S-phase, and stopped upon reaching G2. In the de novo pathway, this phase-dependent synthesis of dNTPs is mediated through the RNR—specifically, through the cell cycle regulation of expression and degradation of the RNR subunit RNR-R2 [117]. In the salvage pathway, the phase-dependent synthesis is controlled by the translational regulation of constituents of the nucleotide salvage pathway [118]. Outside of S-phase, a dNTP hydrolase called SAMHD1 (SAM and HD domain-containing deoxynucleoside triphosphate triphosphohydrolase 1) depletes the dNTP pools by its hydrolase activity to block viral replication, amongst other things [119]. In response to DNA damage, however, dNTP levels can increase by up to 4-fold [96]. In this case, both the RNR and SAMHD1 are then recruited to the site of damage to tightly regulate the amount of dNTPs supplied to the DNA repair machinery [96,120].

Not only replication of the nuclear genome requires a balanced dNTP pool. Unlike the replication of the nuclear genome, the replication of mitochondrial DNA is not regulated by the cell cycle, but is carried out continuously in both mitotic and post-mitotic cells and tissue. Imbalance of the mitochondrial dNTP pools affects the replication of mtDNA, resulting in the accumulation of point mutations and deletions [121,122].

The dNTP pool of the mitochondrial compartment is somewhat separate from the much larger pool supplying the nuclear genome [123,124]. However, cytosolic de novo synthesis of dNTP is essential for mtDNA maintenance, even in post-mitotic cells and tissue [125,126,127].

P53R2 is a protein that substitutes RNR-R2 in post-mitotic cells in response to DNA damage. P53R2 is transcribed by P53 and results, when forming the complex, in the activation of RNR and the de novo synthesis of dNTPs intended as substrates for DNA repair mechanisms [128]. Mutations of the RRM2B gene encoding P53R2 have been shown to induce mtDNA replication and repair deficiency in post-mitotic, but not dividing, human fibroblasts [125]. In humans, mutations in the gene encoding the RRM2B subunit have been correlated with severe mtDNA depletion of muscle tissue, and *RRM2b*^−/−^ mice further display a severe decrease of mtDNA content in liver, kidney, and muscle [126].

It is important to realize that balanced dNTP pools for mtDNA maintenance do not need to be of mitochondrial origin. Imbalances of the nuclear dNTP pools resulting from genetic predisposition, age, or even just diet [129] have the potential to start a vicious cycle, whereby failure to maintain mtDNA integrity results in the decreased synthesis of pyrimidines and further dNTP pool imbalance.

Mitochondrial dysfunction is therefore not only a risk to the mitotic cell, but also fully differentiated post-mitotic cells [130], and is involved in the etiology of a wide array of pathologies, including cancer and Alzheimer’s disease [131,132,133].

## 9. Conclusions

According to recent advancements, hallmarks of cancer include genomic instability, dysregulation of cellular energetics, and mitochondrial dysfunction, which also are common pathways important for cellular aging. Mitochondrial dysfunction is associated with a poor prognosis of many types of cancer, which could very well be linked to an imbalance of the cytosolic dNTP pools, as both of these conditions are related to one of the hallmarks of cancer—chromosomal instability. A better understanding of these pathological cellular processes would advance the development of therapeutic modalities in the prevention of cancer and at the same time help the understanding of biological aging.
